# Incidence, risk factors and outcomes of surgical site infections among patients admitted to Jimma Medical Center, South West Ethiopia: Prospective cohort study

**DOI:** 10.1016/j.amsu.2021.102247

**Published:** 2021-03-29

**Authors:** Gemedo Misha, Legese Chelkeba, Tsegaye Melaku

**Affiliations:** a Department of Pharmacy, Arsi University, Assella, Oromia, Ethiopia; b Department of Pharmacology and Therapeutics, College of Health Sciences, Addis Ababa University, Addis Ababa, Ethiopia; c Department of Clinical Pharmacy, School of Pharmacy, Jimma University, Jimma, Oromia, Ethiopia

**Keywords:** Surgical site infection, Incidence, Risk factors, Outcomes, Ethiopia

## Abstract

**Background:**

Surgical site infections are one of the leading health care–associated infections in developing countries. Despite improvements in surgical technique and the use of best infection prevention strategies, surgical site infections remained the major cause of hospital acquired infections. Therefore, this study aimed to determine incidence, risk factors, and outcomes of surgical site infections among patients admitted to Jimma Medical Center, South West Ethiopia.

**Methods:**

A hospital based prospective cohort study design was employed to follow adult patients admitted to general surgery ward, orthopaedic ward and gynaecologic/obstetrics ward of Jimma Medical Centre, from April 20 to August 20, 2019. All patients were followed daily before, during and after operation for 30 days to determine the incidence of surgical site infection and other outcomes. Data was entered using EpiData version 4.2 and analyzed using statistical software package for social science version 20.0. To identify the independent predictors of outcome, multiple stepwise backward cox regression analysis was done. Statistical significance was considered at p-value <0.05.

**Results:**

Of total of 251 participants included to the study, about 126 (50.2%) of them were females. The mean ± SD age of patients was 38 ± 16.30 years. Considerable number of patients 53(21.1%) developed surgical site infections. American Society of Anaesthesiologists score ≥3 [ Adjusted Hazard Ratio (AHR) = 2.26; 95%CI = (1.03–4.93)], postoperative antibiotic prescription [AHR = 3.2; 95%CI = (1.71–6.01)], contaminated-wound [AHR = 7.9; 95%CI = (4.3–14.60)], emergency surgery [AHR = 2.8; 95% CI = (1.16–6.80)], duration of operation ≥ 2 h [AHR = 4; 95% CI = (2.17–7.50)] and comorbidity [AHR = 2.52; 95%CI = (1.28–4.94)] were independent predictors for surgical site infections. Twenty nine (11.6%) patients returned to operation room. The result of the multivariate cox regression analysis showed that SSI [AHR (95% CI) = 7(3.16–15.72)], and incision site [AHR (95% CI) = 2.5(1.14–5.42)] had statistically significant association with re-operation

**Conclusion:**

The incidence of surgical site infection was high in the study setting. There were significant numbers of contributing factors for the occurrence of surgical site infections. Although no mortality observed during the study period, significant number of patients re-operated. Large multicenter study is urgently needed to confirm the outcome of this study.

## Highlights

• Surgical site infections are one of the leading health care–associated infections in developing countries.
• Considerable number of risk factors are associated with the occurrence of surgical site infection.
• Surgical site infection is associated with increased length of hospital stay and number of patients re-operated.

## 1 Introduction

Surgical site infection (SSI) is defined as an infection that occurs in surgical patients at the incision site within 30 days after surgery if there is no implant or within one year if there is an implant [[1], [2], [3]]. It is a potential complication associated with any type of surgical procedure [4,5]. Epidemiological evidence suggests that approximately 2–5% of surgical patients worldwide have developed surgical site infections [6,7]. However, the incidence is different among developed and developing countries; more patients from developing countries have affected than those from developed countries. The incidence of SSI was 2.6% in United States of America (USA), 1.6% in Germany and 2.9% in different settings of European countries [Italy]. However, in developing country, it accounts two times higher than developed country [8]. The average incidence of SSI in China was 4.5% among patients underwent abdominal surgery [9]. In Sub-Saharan Africa, various study results showed that SSI rate is ranging from 11 to 18% [[10], [11], [12]]. More specifically, systematic reviews and meta-analyses in Ethiopia also showed that the rate of SSI ranged from 12.3% to 25.22% [6,13]. Surgical site infections also varied according to the type of the procedures, where the highest risk was observed for orthopaedic followed by cardiac and intra-abdominal surgery [14,15].

Currently, available data suggest that SSIs increase the length of hospital stay, readmission rate, morbidity, mortality, and financial burdens for individuals and communities. Patients with an SSI have approximately 7–11 additional postoperative hospital-days, 2–11-times higher risk of death [16,17]. Patients who develop SSIs are up to 60% more likely to spend time in an intensive care unit, 5 times more likely to be readmitted and 2 times more likely to die [18]. In USA, it accounts 33.7% of overall hospital-related annual costs and additional 11 days of hospitalization per patient [19]. Established risk factors for SSIs include age, gender, immune status, perioperative hyperglycaemia, pre-existing diabetes, obesity, malnutrition, recent tobacco use, pre-existing remote body site infection, colonization with microorganism, perioperative hypothermia, the improper use of antibiotics and inappropriate pre and intra-operative techniques, type of wound, emergency surgical procedure and prolonged duration of surgery [[20], [21], [22], [23]]. Microorganisms from the patient's own skin flora or from the environment surrounding the patient were the causes of SSIs [[24], [25], [26]]. In both cases, there is a possibility for microorganisms to adhere on surgical instruments and consequently contaminate the incision wound, particularly during contaminated surgical procedures. Most of these infections are caused by multidrug resistant microorganisms [27,28].

There were different studies carried out in Ethiopia on SSIs. The majorities of these were retrospective and cross sectional in design. There was also scarcity of data published in the country regarding patients outcomes related to SSIs. To our knowledge, this is the first study to assess surgical SSIs combined with outcomes at 30 day. Therefore, this was a prospective cohort study aimed to assess the incidence, risk factors and outcomes of surgical site infections at tertiary care hospital.

## 2 Methods

### 2.1 Study setting and period

The Study was conducted at Jimma Medical Centre (JMC); the only teaching and referral hospital in the South Western part of Ethiopia. It is located in Jimma town, 352 km away from the capital, Addis Ababa. It provides services for the catchment population of about 20 million people. Jimma Medical Center has been providing services for approximately 15,000 inpatients, 160,000 outpatients, 11,000 emergency cases and 4,500 deliveries in a year. It has around 1000 beds with 21 clinical service units. Surgery department has about 286 beds. It has different subunits such as general surgical, gynaecology/obstetric and orthopaedics units. The study was conducted from April 20 to August 20, 2019. Ethical clearance was obtained from Jimma University, institute Health institutional review board (IRB) (reference number: IHRPGD/585/2019). We obtained permission from Hospital management before starting data collection. Written informed consent was sought from each participant prior to data collection and informed consent statement was obtained from the legally authorized representatives of illiterate population. We kept the participant's information confidential. Patients who developed SSIs were treated according to the protocol of the hospital. Furthermore, the study was conducted in accordance with the Declaration of Helsinki. The work has also been reported in line with the STROCSS criteria [29].

### 2.2 Study design and population

This was a prospective study that included 251 patients admitted to the surgery wards. The study population for the current study was all adult patients (age ≥ 18 years) who underwent either elective or emergency surgical procedures at general surgery gynecology/obstetric and orthopaedics wards. Patients who had initial diagnosis of SSIs and underwent surgery involving permanent implants were excluded.

### 2.3 Sampling size determination and sampling technique

The sample size was calculated using a single population proportion formula by considering 95% confidence level, 5% margin of error and 19.1% estimated proportion of surgical site infections among patients underwent surgery in Ethiopia [30] and considering 10% non-response rate.

$$n=\frac{{\left(\frac{\text{Z}\alpha }{2}\right)}^{2}\text{*p*}\left(1-\text{p}\right)}{d^{2}}$$

Where: n = desired sample sizes, Zα/2 = critical value or normal distribution at 95% CI which equals to 1.96 (z -value at α = 0.05), P = proportion of patients underwent surgery who developed SSI, D = margin of error (0.05) and therefore, n = (1.96)^2^ × 0.191(1-0.191) ∕ (0.05)^2^ = 237. The number of source population (N) in the study area was the total number of patients who admitted to surgical ward within last 4 months in Jimma Medical Center from September 1, 2018 was 2019. This was obtained from health management information system of surgery department. The size of the population was less than 10,000. Therefore; the sample size was corrected using the correction formula, n_f_ = n × N ÷ n + N = 237 × 2091 ÷ 237+2091 = 210. The calculated sample size; by using the above correction formula was 210. When 10% of non-response added, the minimum adjusted sample size was = 231.

### 2.4 Data collection

In the current study, all consecutive patients meeting the inclusion criteria were recruited. The structured and pretested questionnaires were used to collect relevant data. Detailed data about age, gender, educational status, marital status, residence area, occupational status, alcohol use, cigarette smoking, chat chewing, herbal medicine, duration of labour, antenatal care (ANC) follow up, comorbidities, blood transfusion, ASA score, type of wound, date of admission and operation, readmission and its date, reoperation & its date, length of hospital stay, duration of procedure, urgency of surgery, data about antimicrobials administration pre and post-surgery and duration of administration of the antimicrobials were collected.

### 2.5 Outcomes measure and validating methods

Incidence of surgical site infection was based on culture positive results and/or physician diagnosis and was calculated as:

$$\text{Incidence }=\frac{\text{number of SSIs detected during the study period}}{\text{total number of procedures}\;\;\;\;\text{included during the study period}}\times 100$$

All patients were evaluated and followed up for 30 days postoperatively starting from the date of operation to determine the incidence of SSI. Reoperation is defined as any subsequent at the same site of operation within the first month after the initial procedure. This was obtained from patient medical chart. Operation at different site from the initial site was not considered.

### 2.6 Data processing and analysis

All collected patient's data were entered into Epi-Data version 4.2 for data cleaning and exported to statistical software package for social science (SPSS) version 20.0 for analysis. Descriptive analysis was performed and results were presented in the form of text, tables and graphs. The survival function for occurrences of SSI was checked by Kaplan-Meier (log-rank test). Multicolleanearity test was performed to check for collinearity between independent variables. Cox regression model assumption of proportional hazards was checked by testing of covariates with time. Variables with p-value < 0.25 in bivariate cox regression were candidate for multivariable cox regression. A multivariable cox regression was performed to identify independent predictors of outcomes. Adjusted hazard ratio was used as a measure of strength of association and p-value < 0.05 declared statistical significance.

## 3 Results

### 3.1 Socio-demographic and behavioral measures

A total of 251 patients were included to the study. About 126 (50.2%) were females. The mean ± SD age of the patients was 38 ± 16.30 years. Most of the patients (213, 84.3%) were married and about 105(41.8%) of them cannot read and write. With regard to occupation, 100(39.4%) of study participants were house wife. Farmer accounted for 94(37.5%). Nearly three fourth of the patients reside in the rural area. Limited number of 5(2%) patients reported to consume alcohol on regular basis. Most of the patents (238, 94.8%) were non-smokers. Almost all of the patients (244, 97.2%) were non-users of herbal medicine whereas, 70(27.9%) of patients were Khat chewers [Table 1].

**Table 1:** Baseline socio-demographic and behavioral characteristics of study participants.

Variables		Frequency	Percentage
Sex	Male	125	49.8
	Female	126	50.2
Age(years)	<60	214	85.3
	≥60	37	14.7
Marital status	Single	36	14.9
	Married	213	84.3
	divorced	2()	0.8
Educational status	Cannot read & write	105	41.8
	Primary	99	39.4
	Secondary	33	13.2
	College/university	14	5.6
Occupation	House wife	100	39.8
	Farmer	94	37.5
	Daily laborer	29	11.6
	Student	13	5.2
	Gov't employee	15	6
Residence	Rural	182	72.5
	Urban	69	27.5
Herbal medicine use	Yes	7	2.8
	No	244	97.2
Cigarette smoking	Non smoker	238	94.8
	Ex-smoker	9	3.6
	Current smoker	4	1.6
Alcohol consumption	Never	233	92.8
	Occasionally	13	5.2
	Regularly	5	2
Khat chewing	Yes	70	27.9
	No	181	72.1

### 3.2 Clinical characteristics, surgical procedure type and medication usage patterns

The majority of surgery (167, 66.5%) were emergent. About 148(59%) of surgical incision site were abdominal. Most of wound type (214, 85.26%) were clean or clean contaminated, whereas only 37(14.74%) patients had contaminated wound. Three fourth of the surgical procedure had taken duration of < 2 h. With regard to patient inclusion profile, about 143(56.97%) were from surgical wards, 39(15.54%) from orthopedic wards and the rest were from gynecology/obstetric wards. Nearly one fourth of patients had extended duration of preoperative hospital stay ≥7 days. Moreover, 45(17.9%) of patients were prescribed new antibiotics or reinitiated former antibiotic after discontinuation [Table 2].

**Table 2:** Baseline clinical characteristics, surgical procedure type and medication usage patterns of study participants.

Variable		Frequency	Percentage
ASA score	<3	240	95.6
	≥3	11	4.4
Comorbidity	Yes	50	20
	No	201	80
Preoperative hospital stay(days)	≤7	190	75.7
	>7	61	24.3
Wards	Elective general surgery	54	21.5
	Emergency general surgery	89	35.5
	Orthopedics	39	15.5
	Gynecology-obstetrics	69	27.5
Urgency of surgery	Scheduled	84	33.5
	Emergent	167	65.5
Duration of surgery(hours)	<2 h	187	74.5
	≥2 h h	64	25.5
Type of wound	Clean or clean contaminated	214	85.3
	Contaminated	37	14.7
Location of surgical site	Extremity	103	41
	Abdominal	148	59
Blood transfusion	Yes	38	15.1
	No	213	84.9
Preterm gestation	Yes	7	13
	No	47	87
Duration of labor ≥ 24 h	Yes	36	66.7
	No	18	33.3
Membrane rupture ≥ 12 h	Yes	20	37
	No	34	63
ANC follow	Yes	49	90.7
	No	5	9.3
Preoperative AMP use	Yes	206	82.1
	No	45	17.9
Duration of AMP	Within 24hrs	40	19.4
	>24hrs	166	80.6
Antibiotic use post-surgery	Yes	45	17.9
	No	206	82.1

ANC- Antenatal care, ASA- American Society of Anaesthesiologists: AMP- Antimicrobial prophylaxis.

### 3.3 Disease comorbidity

**Fig. 1:**
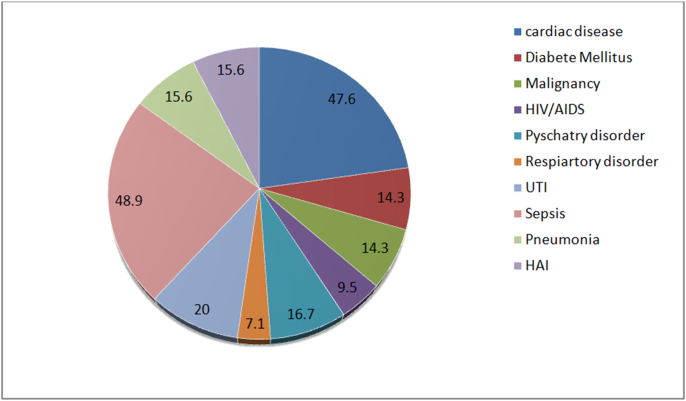
Percentage of disease comorbidity among study participants. ^HIV/ADIS− Human immune virus/Acquired immunodeficiency disease, UTI− urinary tract infection, HAI−Hospital acquired infection^.

From included study participants, 50(20%) of patients were presented with one or more disease co-morbidities. From comorbidities cardiac problem accounted for 47.6%, diabetic mellitus for 14.3%, malignancy for 14.3%, HIV/AIDS for 9.5%, psychiatry problem for 16.7%, and respiratory disorder for 7.1% [Fig. 1].

### 3.4 Incidence of SSIs

**Fig. 2:**
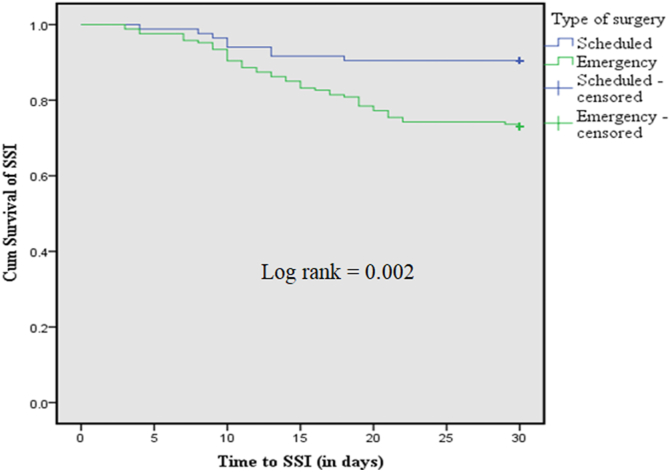
Kaplan-Meier survival curve showing 30 days cumulative survival of SSI for types of surgery among study participants.

A total of 251 patients were followed for 6651 person days. Over this follow up period about 53 patients were develop SSI. The overall incidence rate of SSI was 43.74 per 100,000 person year with 95% CI [33.41–57.25]. The overall proportion of SSI was 21.1% (53/251), of which 49(92.45%) were detected at initial hospitalization while, 4(7.55%) SSIs were confirmed during readmission within 30 days of follow up period. The Kaplan Meier survival curve showed that there was significance difference between scheduled surgery and emergency surgery in terms of time to SSI (log rank = 0.002). The scheduled had an estimated mean survival time of 28 days while emergent surgery had 26 days [Fig. 2].

### 3.5 Factors associated with SSIs occurrences

The association of independent variables with the dependent variable was investigated using both bivariate and multivariate cox regression techniques. On bivariate cox regression analysis, male gender, marital status, residence, ASA score ≥3, ward type, emergent surgery, contaminated wound, duration of surgery ≥ 2 h, absence of preoperative antibiotics prophylaxis, duration of prophylactic antibiotics >24 h, postoperative antibiotics, presence of one or more co-morbidities had statistically significant association with SSI occurrence.

The result of the multivariate cox regression analysis showed that ASA score ≥3 [AHR (95% CI) = 2.26(1.03–4.93)], emergency surgery [AHR(95%CI) = 2.81(1.16–6.80)], contaminated wound [AHR (95% CI) = 7.91(4.29–14.60)], duration of surgery ≥2 h [AHR (95% CI) = 4.03(2.17–7.50)], postsurgical antibiotic prescription [AHR (95% CI) = 3.21(1.71–6.01)] and presence of one or more co morbidity[AHR = 2.52(1.28–4.94)] showed statistically significant association with SSI. The likelihood of SSI occurrence was about 2.26 times more likely among ASA score **≥** 3 patients compared to patients with ASA< 3. Similarly, the relative risk of SSI occurrence was about 2.81 times more likely among patients who underwent emergency surgical procedure than those underwent scheduled surgery. Moreover, patients who had contaminated wound were 7.91 times more likely to develop SSIs compared to patients who had clean or clean-contaminated. The relative risk of SSI occurrence was also higher among patients with duration of operation ≥2 h. Furthermore, patients whose antibiotic was administered postsurgical procedure were 3.21 times more likely to develop SSI [Table 3].

**Table 3:** Cox regression analysis for factors associated with surgical site infections.

Variables		Surgical site infection		P value	CHR (95% CI)	P value	AHR(95% CI)
		Yes	No				
Gender	Male	34(64.15)	91(45.96)	0.028	1.88(1.07–3.29)	0.217	1.5(0.79–2.87)
	Female	19(35.85)	107(54.04)		1		1
Age (years)	<60	42(79.8)	172(86.9)		1		
	≥60	11(20.2)	26(13.1)	0.471	1.6(0.84–3.2)		
Marital status	Single	12(22.64)	24(12.12)		1		1
	Married	40(75.47)	173(87.37)	0.069	0.55(0.29–1.05)	0.06	0.5(0.25–1.01)
	Divorced	1(1.89)	1(0.51)	0.719	1.46(0.19–11.19)	0.915	0.9(0.11–7.55)
Residence	Rural	10(18.87)	59(29.80)	0.129	1.71 (0.86–3.39)	0.317	0.66(0.30–1.5)
	Urban	43(81.13)	139(70.20)		1		
Cigarette smoking	Non smoker	51(96.20)	187(94.44)		1		
	Ex-smoker	1(1.89)	8(4.04)	0.451	0.47 (0.07–3.38)		
	Current smoker	1(1.89)	3(1.52)	0.892	1.15 (0.16–8.30)		
Alcohol consumption per day	Never	47(88.68)	186(93.94)		1		
	Occasionally	4(7.55)	9(4.55)	0.401	1.55 (0.56–4.30)		
	Regularly	2(3.77)	3(1.52)	0.308	2.09 (0.51–8.61)		
Khat chewing?	Yes	14(26.42)	56(28.28)	0.723	0.90 (0.49–1.65)		
	No	39(73.58)	142(71.72)		1		
American Society of Anesthesiologists score	<3	44(83.02)	196(99)		1		1
	≥3	9(16.98)	02(1)	<0.001	6.44(3.13–13.27)	0.041	2.26(1.03–4.93)
Preoperative hospital stay (days)	≤7 days	43(81.13)	147(74.24)		1		
	>7 days	10(18.87)	51(25.76)	0.356	0.72(0.36–1.44)		
Blood transfusion	Yes	9(16.98)	29(14.65)	0.646	1.18 (0.58–2.42)		
	No	44(83.02)	169(85.35)		1		
Wards	Elective	4(7.55)	50(25.25)		1		1
	Emergence	23(43.40)	66(33.33)	0.014	3.8(1.31–10.97)	0.821	0.81(0.13–5.1)
	Orthopedics	19(35.85)	20(10.1)	<0.001	7.9 (2.68–23.20)	0.405	2.2(0.35–13.3)
	Gynecology and obstetric	7(13.21)	62(31.31)	0.591	1.40 (0.41–4.78)	0.666	0.6(0.085–4.83)
Preterm gestation	Yes	1(16.7)	6(12.5)	0.783	0.74 (0.09–6.34)		
	No	5(83.3)	42(87.5)		1		
Duration of labor ≥24hr	Yes	5(83.3)	31(64.6)	0.398	0.40 (0.05–3.39)		
	No	1(16.7)	17(35.4)		1		
Duration of rupture ≥12hr	Yes	3(50)	17(35.4)	0.484	0.57 (0.11–2.80)		
	No	3(50)	31(64.6)		1		
Urgency of surgery	Scheduled	8(15.09)	76(38.38)		1		
	Emergent	45(84.91)	122(61.62)	0.004	3.05 (1.44–6.46)	0.022	2.81(1.16–6.80)
Duration of surgery (hours)	<2	29(54.72)	158(79.80)		1		
	≥2	24(45.28)	40(19.20)	<0.001	2.79 (1.63–4.80)	<0.001	4.03(2.17–7.50)
Type of wound	Clean or clean contaminated	25(47.17)	189(95.45)		1		
	Contaminated	28(52.83)	9(4.55)	<0.001	13.4(7.72–23.27)	<0.001	7.91(4.29–14.60)
comorbidity	Yes	16(30.20)	34(17.17)	0.045	1.82 (1.01–3.27)	0.007	2.52(1.28–4.94)
	No	37(69.80)	164(82.83)		1		
Location of surgical site	extremities	27(50.94)	76(38.38)		.1		
	Abdominal	26(49.06)	122(61.62)	0.129	0.66 (0.38–1.13)	0.60	1.2(0.62–2.32)
Use of prophylactic antibiotic	Yes	50(94.34)	156(78.79)		1		
	No	3(5.66)	42(21.21)	0.022	0.26 (0.08–0.83)	0.656	0.83(0.012–1.03)
duration of antibiotic prophylactic (hours)	Within 24	3(6)	37(23.7)		1		
	>24	47(94)	119(76.3)	0.015	4.3 (1.33–13.71)	0.970	0.98(0.26–3.73)
Use of antibiotic post-surgery	Yes	23(43.40)	22(11.11)	<0.001	4.45 (2.58–7.68)	0.001	3.21(1.71–6.01)
	No	30(56.60)	176(88.89)		1		1

### 3.6 Outcomes: Length of hospital stay, reoperation, readmission and mortality

**Fig. 3:**
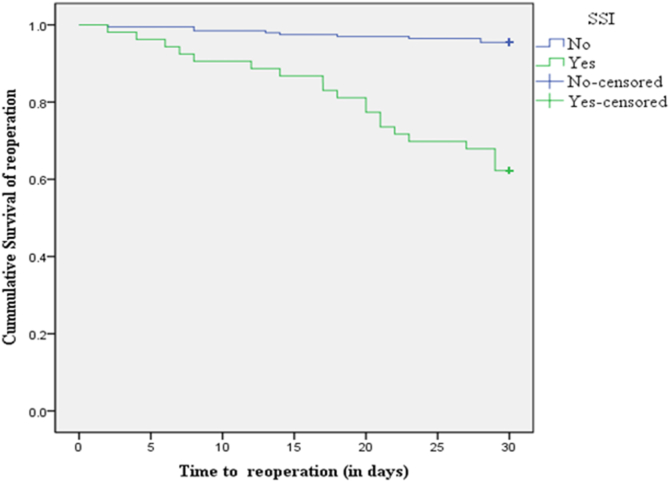
Kaplan-Meier survival curve showing 30 days survival for SSI and reoperation among study participants.

Thirty days outcomes of study participants were also evaluated. Overall, the finding of the present study revealed that the mean ± (SD) length of hospital stay was 14.4 ± 9.7 days for patients developed surgical site infection. About 45(17.93%) patients were remained in hospital until the end of study period. Twenty nine (11.6%) patients returned to operation room and 8(3.88%) patients were readmitted after their initial discharge. However, there was no death registered among the included study participants during the study period. The Kaplan Meier survival curve showed that there was significant difference between patients who developed SSI versus those who did not develop SSI in terms of the time to reoperation(Log rank < 0.0001) Patient with SSI had an estimated mean survival time of 25 days while those without SSI had 29 days [Fig. 3].

### 3.7 Factors associated with Re-operation

On bivariate cox regression analysis, male gender, residence, ASA score ≥3, incision site, contaminated wound, absence of preoperative antibiotics prophylaxis, duration of prophylactic antibiotics >24 h, post-operative elevated white blood cells and SSI had statistically significant association with reoperation. The result of the multivariate cox regression analysis showed that SSI [AHR = 7 (95% CI, (3.16–15.72))] and incision site [AHR = 2.5 (95% CI, (1.14–5.42))] had statistically significant association with re-operation [Table 4].

**Table 4:** Cox regression analysis for factors associated with reoperation.

Variable		Reoperation		P-value	CHR(95% CI)	P-value	AHR(95% CI)
		Yes	No				
**SSI**	Yes	20(37.74)	33(62.26)	<0.001	10 (4.5–21.7)	<0.001	7(3.16–15.72)
	No	09(4.55)	189(95.45)	1	1		1
Age(years)	<60	24(82.8)	190(85.6)		1		1
	≥60	5(17.2)	32(14.4)	0.695	1.2(0.46–3.2)		
Incision site	Extremity	19(65.5)	84(37.8)	0.007	2.87(1.3–6.2)	0.023	2.5(1.14–5.42)
	Abdominal	10(34.5)	138(62.2)		1		1
Duration of surgery(hours)	<2	21(72.4)	166(74.8)		1		
	≥2	8(27.6)	56(25.2)	0.734	1.15(0.51–2.6)		
Urgency of surgery	Scheduled	7(24.1)	77(34.7)				
	Emergent	22(75.9)	145(65.3)	0.257	1.64(0.7–3.83)		
Duration of AMP (hours)	≤24	1(3.4)	39(22.2)		1		1
	>24	28(96.6)	137(77.8)	0.052	7.2(0.98–53.1)	0.274	3.1(0.41–23.3)
ASA score	<3	26(89.7)	214(96.40)				1
	≥3	3(10.3)	8(3.6)	0.117	2.6(0.79–8.6)	0.260	0.5(0.14–1.7)
Comorbidity	Yes	6(20.7)	44(19.8)	0.942	1.03(0.4–2.5)		
	No	23(79.3)	178(80.2)		1		
Gender	Male	18(62.1)	107(48.20)	0.162	1.7(0.81–3.61)	0.125	0.53(0.23–1.2)
	Female	11(37.9)	115(51.8)		1		1
Type of wound	Clean or clean-contaminate	17(58.6)	197(88.7)		1		1
	Contaminated	12(41.4)	25(11.3)	<0.001	4.7(2.23–9.80)	0.739	1.2(0.49–2.73)
Residence	Urban	2(6.9)	67(30.2)		1		
	Rural	27(93.1)	155(69.8)	0.021	5.4(1.29–22.86)	0.150	2.91(0.68–12.4)
AMP use	Yes	29(100)	177(79.7)				
	No	0	45(20.3)	0.103	0.04(0.001–2)	-	-
Khat chewing	Yes	7(24.1)	63(28.4)	0.579	0.8(0.34–1.84)		
	No	22(75.9)	159(71.6)				
Preoperative hospital stay (days)	<7	22(75.9)	168(75.7)				
	≥7	7(24.1)	54(24.3)	0.988	1(0.43–2.40)		
Postoperative WBC	Normal	17(58.6)	166(80.6)		1		1
	Low	1(3.4)	6(2.9)	0.712	1.5(0.2–11)	0.474	0.46(0.05–3.9)
	High	11(37.9)	34(16.5)	0.008	2.8(1.3–5.98)	0.643	1.2(0.53–2.8)

NB-SSI-surgical-site-infection, WBC-white-blood-cell, AMP-antimicrobial-prophylaxis, ASA- American Society of Anaesthesiologists.

## 4 Discussion

Surgical site infections are one of the serious complications of surgical procedures and preventable type of healthcare-associated infections (HAIs) [16,17,27]. They are the leading HAIs reported from low and middle income countries (LMICs) and represent a significant burden in terms of patient morbidity, disability, mortality and additional costs to the health care systems and service payers throughout the world [31,32]. Patients who develop SSIs are up to 60% more likely to spend time in an intensive care unit, 5 times more likely to be readmitted and 2 times more likely to die [18].

In this study, 21.1% of the patients had developed surgical site infection. The finding was in line with other studies done in Ethiopia at Hawassa university referral hospital (19.1%) [30], St Paul's Hospital Millennium Medical college (23.3%) [33], Tikur Anbessa Specialized Hospital (20.6%) [34]. It was also consistent with a retrospective follow-up study included 365 patients that underwent colorectal resection to determine the rate of SSI in USA (23%) [35]. However, the finding was higher than previous studies done at different settings of Ethiopian hospitals; from Lemlem Karl hospital (6.8%) [36], Assella (9.4%) [37], Jimma University Specialized Hospital (11.4%) [38] and Ayder comprehensive specialized hospital (11.7%) [39]. The differences might be due to these studies included only caesarean section. Our founding was also higher than a prospective follow-up study conducted to determine the incidence of SSI in India (5%) [40]. This might be due to exclusion of orthopaedics & Gynecology/obstetric surgery in Indian study. Incidence of present study was lower than the previous reports from Tanzania (25%) [41] and Nigeria (27.56%) [42]. The difference might be due to the study setting and types of study participant's condition; for example, most of patients include to the study in Nigeria had dirty wound.

The incidence of SSI in the current study was also higher than several reports from high and middle income nations. For instance, meta-analyses of observational studies in USA [8],and China [9] showed that the rates of SSI were 2.6% and 4.5%, respectively and a retrospective follow up study in Brazil showed a rate of 3.4% [43].The observed differences in low income countries like Ethiopia might be related to lack of equipment and materials necessary to maintain strict aseptic conditions, poor hygiene of patients, increasing colonization of skin by bacterial flora, late presentation of patients to healthcare system leading to contaminated wounds, and overwhelmed emergency services due to population burden.

The present study found that ASA score **≥** 3, type of wound, duration of surgery, type of surgery, postsurgical antibiotic prescription and presence of comorbidity were significant associated with SSI. Accordingly, The likelihood of SSI occurrences among patients with ASA score of ≥3 were increased by 2.3. These results were consistent with other previous studies [10,43]. This might be due to higher ASA score leads to a worsening of the general clinical status of the patient, prolonging duration of surgery and making it more susceptible to infections.

Patients who had contaminated wound class were more likely to develop SSI as compared with patients who had clean & clean contaminated wound. This result was also supported by many previous other findings [10,34,38,41,44,45]. . Other variable found to be associated with a high incidence of SSIs was duration of surgery ≥ 2 h.The risks of developing SSI among patients whose duration of surgery ≥2 h was 4 times more likely than those with shorter duration. This was in line with many other studies [10,30,34,41,44]. This might be due to a prolonged exposure of tissue to the environment, prolonged hypothermia and declining levels of antibiotics or a greater chance of breach of the aseptic technique in the procedure.

The type of surgery was also statistically associated with SSI in the present study. Being undergoing emergency surgery showed approximately 3 times in the chances of acquiring SSIs when compared to elective surgery. The type of surgery was showed significant association in other literatures [10,45]. This might be due to inadequate preoperative preparation, lack of proper control of other medical co morbidities, and higher risks for contamination in emergency surgeries. Another independent risk factor was post-surgical prescription of antibiotic. Patients who were prescribed new antibiotic or reinitiating discontinued antibiotic after surgery were about 3 times more likely to develop SSIs compared to patients who were not prescribed new or reinitiated antibiotics. This might be due to broad-spectrum and long duration of antibiotic treatment could increase the risk of super-infection. Due to the fact that unrelated infections for which the antibiotic was originally taken, the antibiotic treatment could possibly disturb the normal flora in the body and creates an opportunity for pathogenic microbes to grow and potentially cause a new infection. In our study, the presence of comorbidity was found to be predicator of SSIs. Patients with comorbidity had 2.5 times more likely to develop SSIs and this was agreed with other study [10,46]. The result suggests that patient comorbidity is the primary driver of infection and poor wound healing.

Patients developed SSIs had longer duration of hospital stay compared to those who did not develop SSIs. The average length of hospital stay for those patients developed SSI in the current study was 14.4 ± 9.7 days, which is similar to study by P.J. Jenks et al., 2014 [47]. This is a very noticeable finding as it is associated with extra costs in a country with staggering economy and health care system. Significant number of patients returned to operation room and readmission indicating that SSIs impose huge amount of economic burden on health care system in developing countries.

### 4.1 Strengths and limitations of the study

To our knowledge, this is the first SSI surveillance study in Ethiopia, which describes the 30-day follow-up surveillance. However, too short study period which could not finalize outcomes related to patient that could possibly observed after the study period and therefore underestimate rates of SSI. Furthermore, this study was done only in Ethiopia and at a single center and might be lacked generalizability.

## 5 Conclusion

The incidence of surgical site infection in study setting was relatively high. The occurrence of surgical site infection was associated with contaminated wound class, longer duration of surgery, presence of co morbidity, ASA score of ≥3, postoperative antibiotic prescription and emergency surgeries. Although no mortality observed during the study period, significant number of patients returned to operation room. Future multicenter studies including large number of patients is needed to confirm our study.

## Compliance with ethical standards

All procedures performed in this study involving human participants were reviewed and ethically approved by the Institutional Review Board (IRB) of Jimma University. This study was conducted following the 1975 Helsinki declaration, as revised in 2008 and its later amendments or comparable ethical standards.

## Informed consent

Informed consent was obtained from all patients and authorized representatives of illiterate population (if applicable).

## Availability of data and materials

The data sets generated during and/or analyzed during the current study are available from the corresponding authors on reasonable request.

## Ethical approval

Ethical approval Institutional approval was obtained from the Institutional Review Board of Jimma University

## Funding

This study was sponsored by 10.13039/501100005068Jimma University, Ethiopia.

## Authors’ contributions

The analysis was conceptualized by GM, LCH. Data collection was managed by GM and LCH and data analysis was conducted by GM. LCH and TM drafted the manuscript. All authors participated in editing, feedback and revisions.

## Registration of research studies

Name of the registry: http://www.researchregistry.com

Unique Identifying number or registration ID: **researchregistry6494**

Hyperlink to your specific registration (must be publicly accessible and will be checked): https://www.researchregistry.com/browse-the-registry#home/registrationdetails/60112c597e0d5e001bb24397/

## Guarantor

LC

## Provenance and peer review

Not commissioned, externally peer-reviewed

## Declaration of competing interest

The authors declare that they have no competing interests.

## References

[1] Bagnall N.M., Vig S., Trivedi P. (2009). Surgical-site infection. Surgery.

[2] Leaper D., Tanner J., Kiernan M. (2013). Surveillance of surgical site infection: more accurate definitions and intensive recording needed. J. Hosp. Infect..

[3] Smith M.A. (2013). Clinical practice guideline surgical site infection prevention. Orthop. Nurs..

[4] Moucha C.S. (2011). Modifiable risk factors for surgical site infection. JBJS.

[5] Kirby J.P., Mazuski J.E. (2009). Prevention of surgical site infection. Surg. Clin..

[6] Shiferaw W.S. (2020). Surgical site infection and its associated factors in Ethiopia: a systematic review and meta-analysis. BMC Surg..

[7] Smyth E. (2008). Four country healthcare associated infection prevalence survey 2006: overview of the results. J. Hosp. Infect..

[8] Allegranzi B. (2011). Burden of endemic health-care-associated infection in developing countries: systematic review and meta-analysis. Lancet.

[9] Fan Y. (2014). The incidence and distribution of surgical site infection in mainland China: a meta-analysis of 84 prospective observational studies. Sci. Rep..

[10] Mukagendaneza M.J. (2019). Incidence, root causes, and outcomes of surgical site infections in a tertiary care hospital in Rwanda: a prospective observational cohort study. Patient Saf. Surg..

[11] Sway A. (2019). Burden of surgical site infection following cesarean section in sub-Saharan Africa: a narrative review. Int. J. Wom. Health.

[12] Ngah J.E., Bénet T., Djibrilla Y. (2016). Incidence of surgical site infections in sub-Saharan Africa: systematic review and meta-analysis. Pan Afr. Med. J..

[13] Birhanu Y., Endalamaw A. (2020). Surgical site infection and pathogens in Ethiopia: a systematic review and meta-analysis. Patient Saf. Surg..

[14] Haque M. (2018). Health care-associated infections–an overview. Infect. Drug Resist..

[15] Cheng H. (2017). Prolonged operative duration increases risk of surgical site infections: a systematic review. Surg. Infect..

[16] Organization W.H. (2018). Implementation Manual to Support the Prevention of Surgical Site Infections at the Facility Level: Turning Recommendations into Practice: Interim Version.

[17] Ban K.A. (2017). American College of Surgeons and Surgical Infection Society: surgical site infection guidelines, 2016 update. J. Am. Coll. Surg..

[18] Gagliardi A.R. (2009). Factors influencing antibiotic prophylaxis for surgical site infection prevention in general surgery: a review of the literature. Can. J. Surg..

[19] Zimlichman E. (2013). Health care–associated infections: a meta-analysis of costs and financial impact on the US health care system. JAMA Intern. Med..

[20] Siddalinga S.P. (2011). Abdominal Surgical Site Infection Incidence and Risk Factors.

[21] Xue D. (2012). Risk factors for surgical site infections after breast surgery: a systematic review and meta-analysis. Eur. J. Surg. Oncol..

[22] Utsumi M. (2010). Age as an independent risk factor for surgical site infections in a large gastrointestinal surgery cohort in Japan. J. Hosp. Infect..

[23] Gottrup F., Melling A., Hollander D.A. (2005). An overview of surgical site infections: aetiology, incidence and risk factors. EWMA J..

[24] Yezli S., Barbut F., Otter J.A. (2014). Surface contamination in operating rooms: a risk for transmission of pathogens?. Surg. Infect..

[25] Akhi M.T. (2015). Antibiotic susceptibility pattern of aerobic and anaerobic bacteria isolated from surgical site infection of hospitalized patients. Jundishapur J. Microbiol..

[26] Ibrahimi O.A., Sharon V., Eisen D.B. (2011). Surgical‐site infections and routes of bacterial transfer: which ones are most plausible?. Dermatol. Surg..

[27] Organization W.H. (2018). Preventing Surgical Site Infections: Implementation Approaches for Evidence-Based Recommendations.

[28] Singh R., Singla P., Chaudhary U. (2014). Surgical site infections: classification, risk factors, pathogenesis and preventive management. Int. J. Pharm. Res. Health Sci..

[29] Agha R. (2019). STROCSS 2019 Guideline: strengthening the reporting of cohort studies in surgery. Int. J. Surg..

[30] Laloto T.L., Gemeda D.H., Abdella S.H. (2017). Incidence and predictors of surgical site infection in Ethiopia: prospective cohort. BMC Infect. Dis..

[31] Bedemariam T. (2019). Incidence and Predictors of Postoperative Surgical Site Infections After Major Surgeries at Debre Tabor General Hospital.

[32] Bardossy A.C., Zervos J., Zervos M. (2016). Preventing hospital-acquired infections in low-income and middle-income countries: impact, gaps, and opportunities. Infect. Dis. Clin..

[33] Engida A. (2016). Types and indications of colostomy and determinants of outcomes of patients after surgery. Ethiopian J. Health Sci..

[34] Argaw N.A. (2017). Assessment of surgical antimicrobial prophylaxis in orthopaedics and traumatology surgical unit of a tertiary care teaching hospital in Addis Ababa. BMC Res. Notes.

[35] Shaffer V.O. (2014). Improving quality of surgical care and outcomes: factors impacting surgical site infection after colorectal resection. Am. Surg..

[36] Gelaw K.A. (2017). Surgical site infection and its associated factors following cesarean section: a cross sectional study from a public hospital in Ethiopia. Patient Saf. Surg..

[37] Mamo T. (2017). Risk factors for surgical site infections in obstetrics: a retrospective study in an Ethiopian referral hospital. Patient Saf. Surg..

[38] Amenu D., Belachew T., Araya F. (2011). Surgical site infection rate and risk factors among obstetric cases of Jimma University Specialized Hospital, Southwest Ethiopia. Ethiopian J. Health Sci..

[39] Wendmagegn T.A. (2016). Magnitude and determinants of surgical site infection among women underwent cesarean section in Ayder comprehensive specialized hospital Mekelle City, Tigray region, Northern Ethiopia. BMC Pregnancy Childbirth.

[40] Pathak A. (2014). Incidence and factors associated with surgical site infections in a teaching hospital in Ujjain, India. Am. J. Infect. Contr..

[41] Kisibo A. (2017). Surgical site infection among patients undergone orthopaedic surgery at Muhimbili Orthopaedic Institute, Dar es Salaam, Tanzania. East Centr. Afr. J. Surg..

[42] Olowo-okere A. (2018). Occurrence of surgical site infections at a tertiary healthcare facility in Abuja, Nigeria: a prospective observational study. Med. Sci..

[43] Infecções, D.S.C., E. Incidência, and Y.P.D.R. Incidencia, Surgical Site Infections: Incidence And Profile Of Antimicrobial Resistance in Intensive Care Unit.

[44] Jan, M. and H.A.N. MJ, Surgical Site Infection in a Coastal Tertiary Care Teaching Hospital.

[45] Mengesha R.E. (2014). Aerobic bacteria in post surgical wound infections and pattern of their antimicrobial susceptibility in Ayder Teaching and Referral Hospital, Mekelle, Ethiopia. BMC Res. Notes.

[46] Korol E. (2013). A systematic review of risk factors associated with surgical site infections among surgical patients. PloS One.

[47] Jenks P. (2014). Clinical and economic burden of surgical site infection (SSI) and predicted financial consequences of elimination of SSI from an English hospital. J. Hosp. Infect..

